# An ANN-Based Smart Tomographic Reconstructor in a Dynamic Environment

**DOI:** 10.3390/s120708895

**Published:** 2012-06-27

**Authors:** Francisco J. de Cos Juez, Fernando Sánchez Lasheras, Nieves Roqueñí, James Osborn

**Affiliations:** 1 Project Engineering Area, Department of Exploitation and Exploration of Mines, University of Oviedo, c/ Independencia No 13, Oviedo 33004, Spain; E-Mail: nievesr@uniovi.es; 2 Department of Construction and Manufacturing Engineering, University of Oviedo, Campus de Viesques, Gijón 33204, Spain; E-Mail: sanchezfernando@uniovi.es; 3 Department of Electrical Engineering, Centre for Astro-Engineering, Pontificia Universidad Católica de Chile, Vicuña Mackenna 4860, Santiago, Chile; E-Mail: josborn@ing.puc.cl

**Keywords:** MOAO, adaptive, optics, neural, networks, reconstructor, Zernike

## Abstract

In astronomy, the light emitted by an object travels through the vacuum of space and then the turbulent atmosphere before arriving at a ground based telescope. By passing through the atmosphere a series of turbulent layers modify the light's wave-front in such a way that Adaptive Optics reconstruction techniques are needed to improve the image quality. A novel reconstruction technique based in Artificial Neural Networks (ANN) is proposed. The network is designed to use the local tilts of the wave-front measured by a Shack Hartmann Wave-front Sensor (SHWFS) as inputs and estimate the turbulence in terms of Zernike coefficients. The ANN used is a Multi-Layer Perceptron (MLP) trained with simulated data with one turbulent layer changing in altitude. The reconstructor was tested using three different atmospheric profiles and compared with two existing reconstruction techniques: Least Squares type Matrix Vector Multiplication (LS) and Learn and Apply (L + A).

## 1. Introduction

Telescopes are basically an optical system which amplifies the angular size of a distant object by using lenses and mirrors to manage the light's wave-front. The light emitted by the target travels through the vacuum of space and penetrates the atmosphere before arriving at the telescope. By passing through the atmosphere, it suffers some distortions caused by a series of optically turbulent layers present at different altitudes and with different relative strengths. This turbulence changes the wave-front's shape and morphology. Therefore, correcting the error induced is necessary to obtain a good image quality.

In order to reconstruct the wave-front and eliminate the aberrations, first we must measure and characterize them. In Adaptive Optics (AO), a special design of wave-front sensor called Shack-Hartmann wave-front sensor (SHWFS) is commonly used for this purpose.

Once characterized, the incoming wave-front is then corrected by applying AO techniques. These techniques are used to reconstruct the aberration of the wave-front in the direction of an astronomical target to be observed. The information is then used to modify the surface of a deformable mirror in order to compensate the aberrations in the wave-front [1].

There are two major tomographic AO techniques currently under development: Multi Conjugate (MCAO) [2,3] and Multi Object Adaptive Optics (MOAO) [4,5]. In MOAO, multiple guide stars distributed all over the field are sampled using wave-front sensors with no connection to a deformable mirror (open loop). With that information the integrated aberration in the direction of each target is reconstructed [4,6].

Here we claim a new method based in Feedforward Neural Networks trained off-line, to reconstruct the wave-front aberration in a target direction using slopes measured by a system of off-axis Shack Hartman wave-front sensors in MOAO. We will demonstrate the competiveness of this new method by comparing the results obtained with two reconstruction techniques.

In Section 2 we will describe the materials and methods used: how the sensor works, two other reconstruction techniques that are currently being applied or developed in the world, the metrics we are using to compare the results and a brief introduction to artificial neural networks. In Section 3 we define the parameters for the experimentation and proceed with the simulation, we present the results and discuss them in Section 4. Section 5 talks about the possible future implementation using hardware neural networks.

## 2. Shack Hartmann Wave-Front Sensor

The Shack-Hartmann Wave-front Sensor is a modification of the Hartmann mask developed in 1900 by Johannes Franz Hartmann for focusing telescopes and optical systems. It was developed out of a need to improve the images taken by ground based telescopes, limited in diameter for contemporary imaging systems.

**Figure 1.**
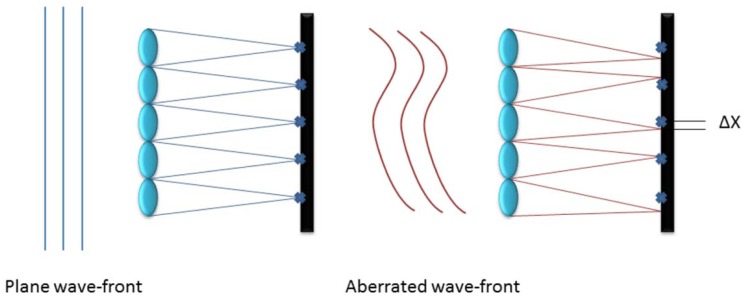
Measurement of wave-front tilts.

Commonly used in astronomy to characterize an incoming wave-front, it consists of an array of lenses with the same focal length (called lenslets) each focused on a photon sensor. This way the incoming wave-front is divided into discrete areas and the local tilt of each lenslet can be measured as the deviation of the focal spot of the sensor from the positions due to a plane wave-front as shown in Figure 1, creating a matrix of tilts characteristic of the wave-front aberration [7]. With this data, the aberration induced in the wave-front by atmospheric turbulence can be approximated in terms of Zernike Polynomials.

**Figure 2.**
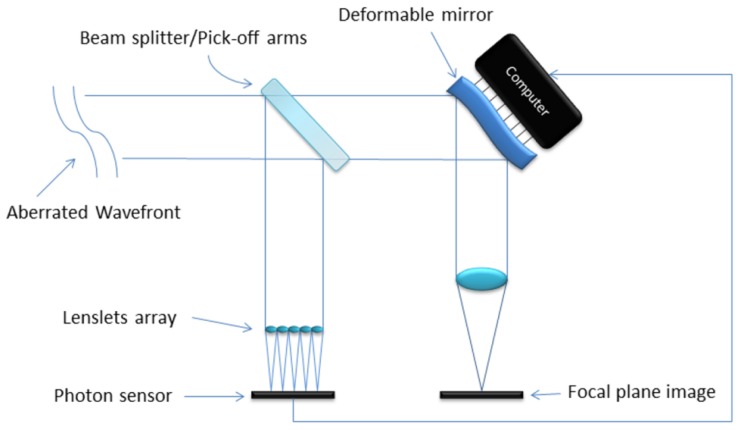
Open loop adaptive optics.

Figure 2 is a simplified schematic of an open loop adaptive optics system. The aberrated wave-front enters the telescope and is split into two streams by a beam splitter (or a pick off arm). One of the new streams is used to measure the wave-front aberration using a SHWFS and shape a deformable mirror in order to correct the second beam and generate the image for observation [7].

As said before, MOAO systems use a number of SHWFS whose fields of view overlap with that of the astronomical target to retrieve aberration information for the whole area of the integrated lightcone to the target. The wave-front aberration is then estimated using a tomographic reconstruction technique. Once done, a deformable mirror is shaped to balance the incoming wave-front and produce a flatter wave input [4,5].

### 2.1. Existing Reconstruction Techniques Based on SHWFS

There are multiple ways to reconstruct the wave-front using the information of different guide stars as sources. The new reconstruction technique will be compared with two of those existing techniques: standard least squares type matrix vector multiplication [8] and learn and apply (L + A) [9].

Learn and apply [9] is a two step reconstruction technique which consist of calculating the covariance matrix that connects the off axis WFS' measured slopes with each other and with one on axis calibration WFS. With both covariance matrices combined, the turbulence profile (strength as a function of altitude) and geometric positions of the guide stars are taken into account in the reconstructor. When the turbulence profile changes, the covariance matrix should be recalculated and hence the system has to stop measuring, since the calibration WFS is not available during observations. However, it is possible to estimate the covariance matrix of the on axis WFS with prior knowledge of the geometry, allowing the system to run even during changeable turbulent conditions, although not at full performance. Also, the open loop WFS can be used to measure the concurrent turbulence profile using the Slope Detection and Ranging (SLODAR) [10,11] method. This way the covariance matrices can be updated when required.

Standard Least Squares type Matrix vector multiplication [8,12] is the standard method for tomographic reconstruction. It consists of a control matrix which, when multiplied by the response of the wave-front sensors, converts the slopes measured into voltages for the actuators. It can be computed off-sky but it is computationally intensive, which represents a problem for future next generation of extremely large telescopes with many wave-front Sensors (WFS) and multiple deformable mirrors (DMs). There is a great interest in avoiding this computational problem.

### 2.2. Reconstruction Performance Metrics

We used two metrics to quantify the optical performance of each system:

- Root Mean Square Wave-front Error (RMS WFE [nm])
- Point Spread Function (PSF) Strehl Ratio

The Root Mean Square Wave-front Error (RMS WFE) is defined as the difference between the average of squared wave-front deviations minus the square of average wave-front deviation. It expresses the statistical deviation from the perfect reference sphere, averaged over the entire wavefront and thus is related to image quality [13]:

(1) $$\text{RMS}=\sqrt{\bar{W^{2}}-{\bar{W}}^{2}}$$

where W is the wave-front deviations.

The PSF Strehl Ratio was introduced by the German astronomer, mathematician and physicist Karl Strehl and is a measure for the optical quality of telescopes and other imaging instruments. It is defined as the ratio between the peak intensity measured in the detection plane and the theoretical peak intensity of the point source with an optical instrument working at the diffraction limit (no aberrations) [13].

For small aberrations can be expressed as:

(2) $$S\equiv e^{-(2\pi \sigma /\lambda )}$$

where λ is the wavelength and σ the Root Mean Square Wave-front Error. It is an indicator of the Peak-Valley error (maximum positive and negative deviations from the desired wave-front) distribution over the wave-front. The Strehl ratio is bound between 0 and 1 with higher values corresponding to a better image quality [13].

## 3. Multilayer Perceptron Neural Network

Artificial Neural Networks are computational models inspired by biological neural networks which consist in a series of interconnected simple processing elements called neurons or nodes [14]. As it is well-known, one of the main advantages of neural networks lays in their ability to represent both linear and non-linear models by learning directly from data measurements [15].

**Figure 3.**
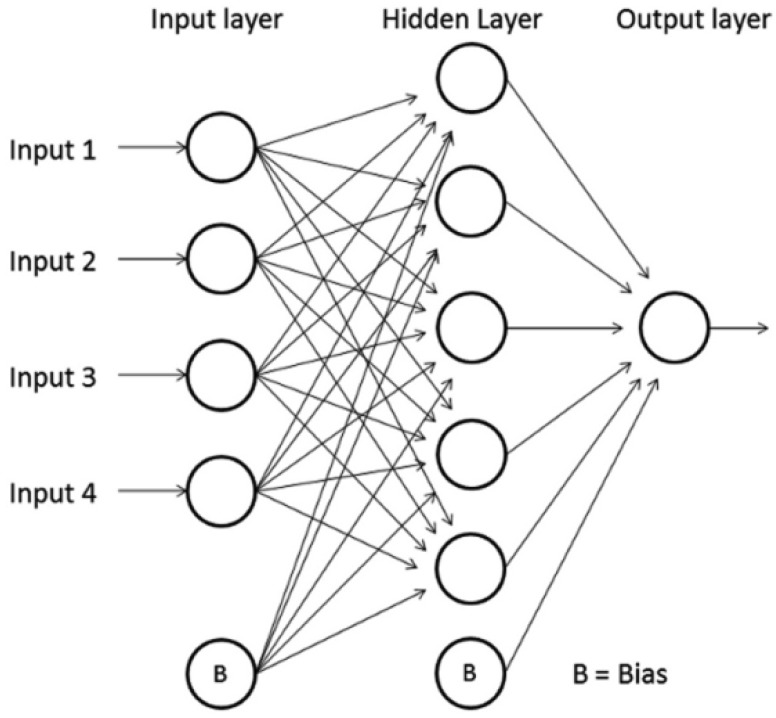
Feedforward MLP neural network.

The Multi Layer Perceptron is a specific type of Feedforward Neural Network. The nodes are organized in layers (input, hidden and output layers) and each neuron is connected with one or more nodes of the following layers only. There is a special type of node called “bias” which has no connection with neurons in the previous layers. Is used to shift the y-intercept value of the activation function for the next layers and therefore enhance the flexibility of the network. Figure 3 shows a scheme of a MLP neural network.

Each neuron receives a series of data (input) from the preceding layer neurons, or an external source, transforms it locally using an activation or transfer function and sends the result to one or more nodes in any of the following layers. This cycle repeats until the output neurons are reached. Each connection between neurons has a numerical value which represents the importance of the preceding neuron in the result of the actual one, called “synaptic weight”, or just weight. It is in these values where the most important fraction of knowledge is stored [16]. Mathematically a neuron of the MLP neural network can be modeled as:

(3) $$Z_{j}(t)=g(S_{i})=g\left(\sum_{i=1}^{n}W_{ji}\cdot Z_{i}(t-1)+W_{j}\right)$$

where Z_j_ is the output of the j^th^ neuron, W_ji_ the synaptic weight between j^th^ and i^th^ neurons, t the actual layer of the Z_j_ neuron, Z_i_ the output of the i^th^ node from the (t−1) layer and W_j_ the bias. The function g(·) is the so called activation function which transforms the input locally.

In supervised learning, training is done by providing representative selection of inputs-desired outputs sets. The weights change to adopt the structure of the function embedded in the data. The way weights are modified to obtain the objective is called “learning algorithm” and is a key feature in the performance of the neural network [17].

### 3.1. Error Backpropagation

The backpropagation error is one of the most popular learning algorithms used for neural network applications. It works by minimizing the square difference between the desired value and the predicted output for all input-output pairs. The square error can be computed as:

(4) $$E=1/2\sum_{i}\sum_{j}{\left(y_{ij}-d_{ij}\right)}^{2}$$

where y_ij_ is the net predicted value, d_ij_ the target is, i goes from 1 to the number of input-output sets and j is an index that represents each output nodes. To minimize E by gradient descent it is necessary to compute its partial derivative with respect each weight [18]:

(5) $$\Delta w_{ij}=-\frac{\partial E}{\partial x_{i}}\cdot \frac{\partial x_{i}}{\partial w_{ij}}=\frac{\partial E}{\partial x_{i}}\cdot \frac{\partial }{\partial w_{ij}}\sum_{k}w_{ik}\cdot y_{k}=\delta_{i}\cdot w_{ij}$$

with w_ij_ connecting the i^th^ neuron with the j^th^. So we have to compute all errors (δ_i_) for all the neurons involved in the net. The algorithm is able to do this in a two steps process:

1^st^ step: Compute the state for all the neurons (hidden included) in the net using Equation (3).
2^nd^ step: Propagate the error backwards until reaching the input units. By applying the chain rule we are able to compute the error of the hidden units in terms of its next layer nodes states, errors and weights as shown by Equation (6):
  (6) $$\delta_{j}=\frac{\partial E}{\partial x_{j}}=-\sum_{i\in P_{j}}\frac{\partial E}{\partial x_{j}}\cdot \frac{\partial x_{i}}{\partial y_{j}}\cdot \frac{\partial y_{j}}{\partial x_{j}}=f^{\prime }\left(x_{j}\right)\cdot \sum_{i}\delta_{i}\cdot w_{ij}$$

by knowing the error of the output nodes we can calculate the errors of the nodes in the preceding hidden layers, and so on until reaching the input neurons. This allows us to compute the negative error gradient for all units in the net.

Usually the weights increase proportionally to the negative gradient computed by the method. The proportional constant is called “learning rate” and is a key parameter of neural network training [16]:

(7) $$\Delta w_{ij}=-\varepsilon \frac{\partial E}{\partial w_{ij}}$$

There are two ways to use the algorithm: one is to change the weights after each input-output set, minimizing the memory needed for the algorithm to work. The other was used in this works and consists in accumulating all the weight gradients for all the data pairs before applying the change. This process of computing weight changes for all the data repeats a number of times defined by the user. Each iteration is called an “Epoch”.

Care should be taken to avoid over training. Depending on the learning rate, neural networks learn faster or slower and so their weights change by higher or lower magnitudes. If the combination of epochs, learning rate and data quality is out of balance, the net can be infra or overtrained, losing performance.

### 3.2. Neural Network Performance Metrics

To monitor the net performance we used three metrics: Root mean square error, normalized error and accuracy. Root Mean Square Error is widely used in regression and situations alike, it measures how much the outputs are deviated from the target values:

(8) $$\text{RMS}E=\sqrt{1/N\sum_{j=1}^{N}{\left(y_{j}-d_{j}\right)}^{2}}$$

where N is the number of outputs, y_j_ the predicted values and d_j_ the target values

The problem with the RMSE is that it is corrupted by the target variances and so cannot be compared with other neural networks working in different situations. The variance is defined as the mean squared deviation from the mean of a variable in a population, as shown by Equation (2):

(9) $$\sigma^{2}=1/M\cdot \sum_{j=1}^{M}{\left(d_{j}-\mu \right)}^{2}$$

It can be applied to the whole net or to each neuron individually in the case of different variances for each node. The normalized error is a measure that removes the effect of the target variance and it is independent of network configurations [19], returning values between 0 and 1. It can be seen as a measure of the output variance that is due to error rather than target variance or networks architecture [19]. To compute the normalized error it is necessary to calculate the sum of squared deviations of the target from its mean (Equation (10)):

(10) $$E_{\text{mean}}=\sum_{j=1}^{N}\sum_{i=1}^{M}{\left(d_{ji}-\mu_{j}\right)}^{2}$$

where N is the number of outputs and M the number of cases. The total squared error of the net is:

(11) $$E_{t}=\sum_{i=1}^{M}\sum_{j=1}^{N}{{\left(y_{j}-d_{j}\right)}_{i}}^{2}$$

Thus, the normalized error is defined as:

(12) $$E_{n}=E_{t}/E_{\text{mean}}$$

The closer to 0 the better, since it means that the pattern is being learnt properly. On the other hand a value of En close to 1 means that the net is returning the mean as the desired output for all input sets. The backpropagation neural networks learn this pattern relatively easily so the normalized error is particularly useful for them [20]. It can be used with the whole net or with each output v ariable separately.

Accuracy is defined as the proportion of correct predictions relative to the size of dataset. It is commonly used in discontinuous output neural networks since it easy to compute the positive and negative values. In the case of a continuous output, a threshold error value must be set to classify the predicted values and be able to compute the accuracy of the net. For this work the threshold value is 5% of the full interval for each output [21].

## 4. Simulation Parameters

The reconstructor is trained to return the first six radial orders of Zernike coefficients (not including the piston) using the wave-front off-axis slopes measured by three SHWFS with an array of 7 × 7 subapertures as inputs. The subaperture slopes can also be used for this purpose, but the computational load for this is higher, increasing the training time so much and impeding a proper evaluation of different net structures and layers distributions. Also, the complexity of the problem increases and the performance of the network may be affected, driving to worse results or to modifications on the network that will bring more computational load to the experiment.

We used a multilayer perceptron with back propagation error as the learning algorithm, tested with a series of network morphologies: one or two hidden layers containing different numbers of neurons (which defines the degrees of freedom), different learning rates and activation functions. The networks architecture depends on the complexity of the problem: more complex situations require network structures with more degrees of freedom. In our case the complexity increases with the number of layers, their altitude and the difference between their properties.

For training we used simulation data, since we can take full control of the input-output sets to feed and avoid outliers, missing data and other measurement problems. The most realistic scenario for simulating is that with multiple turbulent layers of different strengths and at different heights. Another option is to simulate a surface dominant layer with a second layer at a number of different heights, which is a simplification of the real turbulent status [22–24].

We have tested many training scenarios, including the ones cited before, with different network morphologies and parameters. Finally, the best performance was achieved by the combination of simulating a single layer placed at 155 altitudes ranging from 0 to 15,500 m. with a 150 m. step for training data and the net parameters present in Table 1. By this way, 1,000 random data sets are generated for each altitude culminating in a 155,000 training set, which includes all possible positions of the layer in the atmosphere. The net itself combines all the possibilities and responses and is capable of estimating the output of much more complex profiles. Table 1 shows a summary of all the parameters used for the training of the neural networks.

Due to the fact that the initial weights of the net are randomized, all the networks show different output values for the same input even if they were trained using the same parameters and data. In order to decrease the variance and enhance the accuracy of the networks, seven different neural networks were trained with the same training set and parameters, and used simultaneously to average their output vectors [25–27].

All the simulations are made assuming three off axis natural guide stars equally spaced in a ring of 30 arcseconds radius working on a 4.2 m telescope. The target position is at the centre of the field of view and all the WFS are Shack-Hartmant WFS with 7 × 7 subapertures, 100 photons and 20 × 20 pixels per subaperture. These conditions are designed to be similar to those of the CANARY experiment in order to compare the results with the on-sky results from the Learn and Apply algorithm.

For testing, we used Monte Carlo simulation to generate three different test cases: good, medium and bad seeing atmospheric profiles, derived from the CANARY experiments in La Palma (Canary Islands, Spain). All have four turbulent layers but the altitude and relative strengths are different in each case. The Learn and Apply and LS techniques were both reconfigured between tests to optimize their prediction capacity, while no change was made to the ANN reconstructor. Table 2 shows the parameters of the turbulent layers in each test.

## 5. Results and Discussion

### 5.1. Neural Network Performance Metrics

Table 3 shows the performance metrics applied to the neural networks with Tests 1, 2 and 3 respectively from output neuron 1 to 5 (Zernike coefficients).

As expected, the RMSE increases from Test 1 to 3 due to the higher complexity of the latters.

Because Test 3 is the hardest atmospheric profile for tomography, due to its strong high altitude turbulence, its variance and interval is higher than in the other test cases. This higher variability implies that the normalized error is lower in the 1^st^ and 2^nd^ Zernike coefficients of Test 3 than in the other tests due to the higher squared difference from the mean that the real values have. The best performance for Test 3 suggests that the net is more able to fit this data than the other test cases, although due to its high complexity the overall error is higher.

There is also a difference between the first order coefficients and the higher orders. With only five orders represented it is easy to find a general trend in each metric used: RMSE decreases with increasing Zernike order, normalized error and accuracy decreases due to higher variance and interval.

### 5.2. Tomographic Metrics

All the results for the reconstructor were compared with the same simulations applied to the LS and the Learn and Apply systems in order to evaluate the difference in performance between them. All reconstructors were applied to a modal DM correcting the same number of Zernike modes for reconstructing the phase. The results are shown in Table 4.

It is clear that ANN was able to handle the three different atmospheric profiles, even when there is no additional information provided between tests. In a real situation, where the atmospheric profile changes with time in an unknown way and speed, other reconstructors may not be able to handle these changes as well a the ANN since they have to be recalibrated. All the metrics used indicate that the best performance is achieved with the ANN reconstructor, followed by the L + A and the LS.

However these three test cases are all similar and so there won't be so much difference in the performance of the reconstructors. We have also applied three unrealistic extreme profiles with two turbulent layers: one at the ground and the other at different altitudes, splitting the turbulence strength equally between those layers. As with the above test cases, LS and L + A techniques were reconfigured for each test while ANN remained unchanged. Table 5 presents the WFE and Strehl ratio with the different atmospheric configurations.

Table 5 shows that ANN has better behavior than the other reconstruction techniques in the three profiles, demonstrating the stability of the reconstructor even in these extremely changeable conditions. In Table 5 can also be noted that the performance of all the reconstructors decreases with increasing altitude of the high layer because the reduced fraction of the lightcone overlapping phenomena [28].

**Figure 4.**
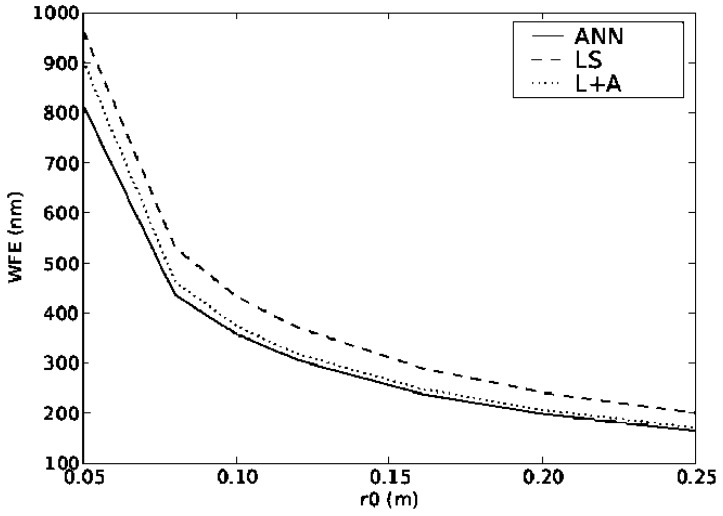
WFE as a function of turbulence strength r_0_ and the outer scale L_0_.

**Figure 5.**
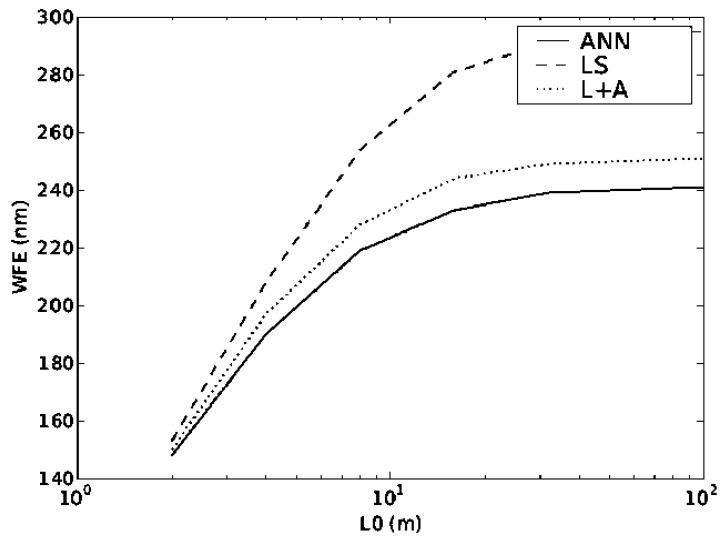
WFE as a function of turbulence strength r_0_ using Test 2 atmospheric profile.

The plots in Figures 4 and 5 show the performance of all of the reconstructors when the values of the turbulence strength r_0_ and outer scale L_0_ changes. It can be seen that even though the ANN was trained with only a single layer changing its altitude, the values of WFE for both dynamic systems are lower than using the other reconstructors reconfigured. It is important that although the ANN was trained with one value of r_0_ and L_0_ it can actually function with quite a large range of input values, covering the full range of expected values in atmospheric turbulence. Again the other two methods were optimised for each particular parameter value.

## 6. Future Implementation: Hardware Neural Networks

An important question for AO instrument scientists is the scalability to ELT size telescopes. Due to the larger number of subapertures and guide stars involved tomography on ELT scales becomes computationally more difficult. Although the training of the ANNs becomes exponentially more time consuming for larger telescopes (or more correctly, for larger number of subapertures) the computational complexity remains constant. Therefore, given enough time a network can be trained and implemented on ELT scale telescopes. Although we think that it might be possible to extrapolate the correction geometrically for any target direction it is worth noting that currently for every different asterism a new training is required. Therefore advanced planning is necessary.

All the ANNs architectures and associated learning algorithms take advantage of the inherent parallelism in the neural processing [29], but for specific applications such as tomographic reconstruction at ELT scales, which demand high volume adaptive real-time processing and learning of large data-sets in reasonable time scales, the use of energy-efficient ANN hardware with truly parallel processing capabilities is more recommended. Hardware devices designed to realize artificial neural network are referred as hardware neural networks (HNN).

Specialized ANN hardware (which can either support or replace software) offers appreciable advantages in these situations [30] and can offer very high computational power at limited price and thus can achieve several orders of speed-up, especially in the neural domain where parallelism and distributed computing are inherently involved. For example, very large scale integration (VLSI) implementations for cellular neural networks (CNNs) can achieve speeds up to several teraflops [29–31], which otherwise is a very high speed for conventional DSPs, PCs, or even work stations.

To address the challenge of mapping highly irregular and non-planar interconnection topology entailing complex computations and distributed communication a wide spectrum of technologies and architectures have been explored in the past. These include digital [32–34], analog [35,36], hybrid [37,38], FPGA based [39–41], and (non-electronic) optical implementations [42–44].

Although not as widespread as ANNs in software, there do exist HNNs at work in real-world applications. Examples include optical character recognition, voice recognition (Sensory Inc. RSC Micro controllers and ASSP speech recognition specific chips), Traffic Monitoring (Nestor TrafficVision Systems), Experiments in High Energy Physics [45] (Online data filter and Level II trigger in H1 electron–proton collision experiment using Adaptive Solutions CNAPS boards), adaptive control, and robotics [31].

Sundararajan and Saratchandran [46] discussed in detail various parallel implementation aspects of several ANN models (back propagation (BP) based NNs, recurrent NN *etc.*) using various hardware architectures including scalable general purpose parallel computers and MIMD (multiple instruction multiple data) with MPI interface. Individual chapters discuss reviews, analysis, and experimental case studies, e.g., on implementations for BP based NNs and associated analysis of network and training set parallelisms.

Since one tomographic reconstructor at ELT scales will use networks with less than 10^5^ neurons and/or inputs and will only need occasional training, software should be sufficient in such situations [30]. But even if ANN algorithms develop to the point where useful things can only be done with 10^6^–10^8^ of neurons and 10^10^–10^14^ of connections between them [47,48], high performance neural hardware will become essential for practical operations. It is important to add that such large scale neural network hardware designs might not be a distant reality as is apparent from the recent work of Schemmel *et al.* on wafer-scale integration of large SNN models [49,50].

Finally, in spite of the presence of expressive high-level hardware description languages and compilers, efficient neural-hardware designs are well known for achieving high speed and low power dissipation when the application involves computational capabilities exceeding of workstations or personal computers available today [31]. We are not able at this point to define the final computational necessities of an ANN tomographic reconstructor at ELT scales but, as an example of the capabilities of a wide implemented HNN, a typical real-time image processing task may demand 10 teraflops_1_, which is well beyond the current capacities of PCs or workstations today. In such cases neurohardware appears attractive choice and can provide a better cost-to-performance ratio even when compared to supercomputers.

Assuming the frame-rate of 100 fps, frame size of 1,280 × 1,024 pixels with 3 bytes per pixel, and average number of basic imaging operation having computational complexity of θ(N), (N is the frame-size) with 10^5^ such operations to be performed on each frame.

## 7. Conclusions

The potential of Artificial Neural Networks in reconstructing the wave-front from measurements from several off-axis Shack Hartmann WFSs has been proven by training a network with a series of datasets designed to cover the full range of possible input vectors. This datasets are obtained by simulating a single turbulent layer changing its altitude, generating a set of different scenarios in which the overlapping effect of the light cones changes.

Some network morphologies and learning algorithms have been tested and used to evaluate its performance, concluding that the best morphology for fitting this data is the simplest one: MLP with the same neurons in the hidden layer than in the input layer to allow full mapping and using continuous sigmoid function as activation function. Other morphologies result in better performance for specific test cases but this one gives a good result in all cases. However, there are still a lot of network morphologies and algorithms that have not been tested in this work, so further investigation in this aspect is possible.

The neural network trained in this work seems to have better performance with highly complex turbulence profiles than with low complexity ones. Although the overall RMSE is smaller with the simpler ones, the fraction of the variance due to the error itself is smaller in the test 3 scenario than in the others. Even so, the net showed a great performance in the other two profiles.

Comparing the ANN reconstructor with other existing and in development techniques like LS and L + A, it is shown that the novel reconstruction technique results in a lower residual WFE and better image sharpness.

One of the major advantages of ANN over other systems is that no re-training is required when the atmospheric profile changes. The network was able to cope with all the turbulent profiles tested in this work with no change during operation.

The most concerning problem for future implementation of the technique is to avoid the computational problem commonly reported in neural networks. With the novel ELT and VLT, more Shack Hartmann wave-front sensors are used and so more input and hidden neurons are needed, increasing the computational load of the machine exponentially. Even when there are some actual computer systems capable of handling this process they are no match in speed, performance and energy consumption for a Hardware Neural Network system that take advantage of the parallelism inherent to the neural networks.

In the future the performance of the technique needs to be tested in a more realistic situation, on a lab bench and on sky.

## Figures and Tables

**Table 1.** Summary of parameters of the neural network.

**Parameter**	**Value**
Neural Network	Multi layer Perceptron
Number of hidden layers	1
Neurons	222(input)-222(hidden)-27 (output)
Activation function	Continuous sigmoid function
Learning algorithm	Backpropagation error
Learning rate	0.01
Epochs	10,000

**Table 2.** Layer parameters of the three test cases.

**Layer**	**Parameter**	**Values**			**Units**
Common	Test name	test1	test2	test3	
	r_0_	0.16	0.12	0.085	m

Layer 1	Altitude	0	0	0	m
	Relative strength	0.65	0.45	0.8	
	Wind Speed	7.5	7.5	10	m/s
	Wind direction	0	0	0	degrees

Layer 2	Altitude	4,000	2,500	6,500	m
	Relative strength	0.15	0.15	0.05	
	Wind Speed	12.5	12.5	15	m/s
	Wind direction	330	330	330	degrees

Layer 3	Altitude	10,000	4,000	10,000	m
	Relative strength	0.1	0.3	0.1	
	Wind Speed	15	15	17,5	m/s
	Wind direction	135	135	135	degrees

Layer 4	Altitude	15,500	13,500	15,500	m
	Relative strength	0.1	0.1	0.05	
	Wind Speed	20	20	25	m/s
	Wind direction	240	240	240	degrees

**Table 3.** Network performance metrics with test 1, 2 and 3.

**Test**	**Metric**	**Coeff. 1**	**Coeff. 2**	**Coeff. 3**	**Coeff. 4**	**Coeff. 5**
Test 1	RMSE	0.8976	0.8464	0.6917	0.6159	0.6303
	Normalized Error	0.0374	0.0345	0.1007	0.0844	0.0765
	Accuracy	94.8	97.13	77.22	80.1	83.63

Test 2	RMSE	1.0445	1.0387	0.7746	0.6891	0.7121
	Normalized Error	0.0314	0.0327	0.0773	0.0661	0.0614
	Accuracy	96.49	95.44	84.52	84.9	86.99

Test 3	RMSE	1.0941	1.0902	1.0082	0.8701	0.9312
	Normalized Error	0.0195	0.0200	0.0743	0.0589	0.0586
	Accuracy	99.29	99.46	85.94	85.91	89.7

**Table 4.** Test results with the three reconstruction techniques.

**Test**	**Technique**	**WFE**	**Strehl ratio**
Test 1	Uncorrected	644	0.048
	LS	293	0.296
	L + A	251	0.402
	ANN	231	0.462

Test 2	Uncorrected	817	0.025
	LS	322	0.23
	L + A	289	0.3
	ANN	262	0.37

Test 3	Uncorrected	1088	0.012
	LS	454	0.068
	L + A	409	0.1
	ANN	387	0.125

**Table 5.** Wave-front Error and Strehl ratio for the three reconstruction at extreme test cases with increasing altitude.

**Reconstructor**	**Altitude of high layer (m)**	**WFE (nm)**	**Strehl ratio**
Uncorrected	5,000	767	0.064
LS		293	0.289
L + A		269	0.353
ANN		211	0.52

Uncorrected	10,000	818	0.025
LS		465	0.066
L + A		372	0.147
ANN		297	0.287

Uncorrected	15,000	815	0.026
LS		574	0.043
L + A		466	0.069
ANN		390	0.127
